# Association of Longitudinal Cognitive Decline With Amyloid Burden in Middle-aged and Older Adults: Evidence for a Dose-Response Relationship

**DOI:** 10.1001/jamaneurol.2017.0892

**Published:** 2017-07-10

**Authors:** Michelle E. Farrell, Kristen M. Kennedy, Karen M. Rodrigue, Gagan Wig, Gérard N. Bischof, Jennifer R. Rieck, Xi Chen, Sara B. Festini, Michael D. Devous, Denise C. Park

**Affiliations:** 1 Center for Vital Longevity, School of Behavioral and Brain Sciences, University of Texas at Dallas, Dallas; 2 Multimodal Neuroimaging Group, Department of Nuclear Medicine, University Hospital Cologne, Cologne, Germany; 3 Institute of Neuroscience and Medicine (INM-3), Cognitive Neuroscience Research Center, Jülich, Germany; 4 Rotman Research Institute, Baycrest Health Sciences, Toronto, Ontario, Canada; 5 Avid Radiopharmaceuticals, a Wholly Owned Subsidiary of Eli Lilly, Philadelphia, Pennsylvania; 6 Department of Psychiatry, University of Texas Southwestern Medical Center, Dallas

## Abstract

**Importance:**

Presently, the clinical standard for reporting the results of an amyloid positron emission tomography scan is to assign a dichotomous rating of positive or negative for the presence of amyloid. In a 4-year longitudinal study, we investigated whether using a continuous measure of the magnitude of baseline amyloid burden would provide valuable information about the rate of future cognitive decline over the subsequent 4 years compared with a dichotomous measure in middle-aged and older adults.

**Objective:**

To examine whether a continuous, dose-response relationship between amyloid burden and cognitive decline was present among middle-aged and older adults.

**Design, Setting, and Participants:**

This cohort study included 174 participants from the Dallas Lifespan Brain Study who were 40 to 89 years old at the beginning of the study, were cognitively normal at baseline (a Mini-Mental State Examination score of 26 or higher) with no history of neurological or psychiatric disorders, and had completed amyloid imaging ([^18^F]-florbetapir) at baseline and cognitive assessments at baseline and a 4-year follow-up. Continuous amyloid burden was measured as the mean cortical standardized uptake value ratio (SUVR) at baseline.

**Main Outcomes and Measures:**

Linear mixed models assessed the effect of increasing baseline amyloid over time (SUVR × time interaction) on episodic memory, reasoning, processing speed, vocabulary, and Mini-Mental State Examination performance. Age, sex, education, apolipoprotein ε4, and the random effect of intercepts were included as covariates.

**Results:**

The mean (SD) age for all participants (n = 174) was 66.44 (11.74) years, and 65 participants (37%) were men. The primary analyses yielded significant SUVR × time interactions in episodic memory, processing speed, vocabulary, and Mini-Mental State Examination performance, but not in reasoning performance. Higher baseline SUVR projected greater cognitive decline over 4 years. When controlling for variance related to a dichotomized positive/negative classification, most effects on cognition remained. Dichotomized amyloid status alone yielded fewer significant effects of amyloid on cognitive decline than continuous SUVR. Among amyloid-positive participants, increasing baseline SUVR predicted an increasing decline in episodic memory, but other effects on cognition were more limited. Finally, higher baseline amyloid burden among middle-aged adults was related to changes in vocabulary, with the effect driven by 3 apolipoprotein ε4 homozygotes.

**Conclusions and Relevance:**

These results suggest that the magnitude of amyloid burden at baseline is associated with the rate of cognitive decline over 4 years and potentially provides important information about the rate of future cognitive decline that is not available from a dichotomous positive/negative categorization.

## Key Points

**Question:**

Is there a dose-response relationship between the magnitude of amyloid burden and the rate of cognitive decline among healthy middle-aged and older adults?

**Finding:**

In this longitudinal cohort study, healthy adults aged 40 to 89 years were assessed with ^18^F florbetapir positron emission tomography imaging at baseline and cognitive measures at baseline and 4-year follow-up. Increasing baseline amyloid burden predicted a steeper decline in episodic memory, processing speed, vocabulary, and Mini-Mental State Examination performance.

**Meaning:**

The initial magnitude of amyloid deposition in healthy adults may be associated with the rate of future cognitive decline and provides important information that is lost when only dichotomous information (positive/negative) is provided.

## Teaser

This longitudinal cohort study explores how the magnitude of baseline amyloid burden predicts the rate of cognitive decline over 4 years in cognitively normal adults while controlling for dichotomized amyloid status.

## Introduction

The advent of in vivo amyloid imaging has resulted in a corpus of cross-sectional data that indicate that cognitively normal older adults vary considerably in their magnitude of amyloid deposition. Those in the higher range of amyloid burden often demonstrate deficits in episodic memory,^1^ a hallmark of Alzheimer disease (AD), and other domains of cognition.^4^ Contemporary models of AD posit that healthy individuals harboring elevated amyloid pathology are in a preclinical stage of AD and on a trajectory toward developing dementia.^10^

Longitudinal studies of an amyloid-cognition relationship in cognitively normal adults are limited, and to date most studies have reported greater rates of episodic memory decline for amyloid-positive adults compared with amyloid-negative adults.^12^ These studies have largely treated amyloid as a categorical variable, classifying individuals as either amyloid-positive or negative based on study-specific thresholds. This dichotomous approach has been valuable for establishing that the presence of suprathreshold amyloid deposition is associated with greater cognitive decline.

However, a dichotomous approach provides limited information about the potential continuous relationship between amyloid burden and cognitive decline. Given that amyloid burden exists on a continuum, increasing baseline amyloid burden may predict a corresponding increase in the rate of future cognitive decline. Furthermore, dichotomous approaches rely on selecting a positivity threshold, with methods varying across studies. A conservative threshold may incorrectly exclude individuals who are actually amyloid-positive, while a more liberal threshold is more likely to include some false positive results. Using a continuous standardized uptake value ratio (SUVR) avoids these issues.

The present approach used data from the Dallas Lifespan Brain Study to evaluate the dose-response relationship between continuous baseline SUVR and cognitive decline over 4 years. In an earlier cross-sectional study^6^ that also used participants from the Dallas Lifespan Brain Study, we reported such a dose-response correlation, with higher amyloid burden associated with slower processing speed and a lower reasoning ability. A recent longitudinal study by Lim et al^16^ divided amyloid-positive participants (including both cognitively normal adults and those with mild cognitive impairment) into higher and lower amyloid burden groups. They reported greater cognitive decline in the higher amyloid group compared with the lower amyloid group, supporting the possibility of a dose-response relationship. The present study differs from Lim et al^16^ and other studies by treating amyloid as a continuous variable, controlling for dichotomized amyloid status, and comparing the results yielded by both continuous and dichotomous approaches. Moreover, we examined whether results differed when amyloid positivity was dichotomized using 2 different thresholds.

A second important feature of this study is the inclusion of both middle-aged and older adults (ages 40-89 years). To our knowledge, most amyloid imaging research to date has focused only on older adults (age 60 years and older). Recently, randomized clinical trials have increasingly targeted middle-aged adults for interventions,^17^ but almost nothing is known about amyloid burden in middle age. Although we found at baseline^6^ that middle-aged adults (ages 40-59 years) had lower amyloid burden than older adults, there was considerable variance in baseline amyloid levels, and we considered the possibility of finding a dose-response relationship between amyloid and cognitive decline as early as middle age.

## Methods

### Participants

The study includes the first 184 Dallas Lifespan Brain Study participants who completed amyloid positron emission tomography (PET) scans, structural magnetic resonance imaging (MRI) scans, and a cognitive battery at baseline and who returned for a 4-year follow-up. A total of 255 participants were eligible to return, of whom 189 returned (retention rate, 74%). Reasons for participants not returning included 7 participants who died, 18 who were in poor health, 14 who were not interested in returning, and 28 who were lost to follow-up. In addition, 3 participants were excluded because their MRI results had poor image quality and 2 were excluded because of computer malfunctions, for a final sample of 184 adults. Those who continued to participate in the study did not differ significantly from those lost to follow-up as a function of age, baseline SUVR, years of education, sex, or apolipoprotein ε (APOE) carrier status (*P* values ranged from .46-.83).

The median (SD) follow-up time was 3.82 (0.32) years. At baseline, all participants had a Mini-Mental State Examination (MMSE) score of 26 or more. At follow-up, MMSE scores were 25 or more. All participants were recruited locally from advertisements and public talks and were screened for neurological and psychiatric disorders, loss of consciousness for more than 10 minutes, a history of drug or alcohol abuse, and having undergone major heart surgery or chemotherapy within 5 years. All were native English speakers and right handed. This study was approved by the University of Texas Southwestern and the University of Texas at Dallas institutional review boards. All participants provided written informed consent and were debriefed according to human investigations committee guidelines.

### Cognition

Five cognitive outcome measures were derived from the cognitive battery. Three were averaged composites: episodic memory (Hopkins Verbal Learning, PAR Inc;^18^ CANTAB Verbal Recognition Memory, Cambridge Cognition^19^), processing speed (Wechsler Adult Intelligence Scale digit symbol;^20^ digit comparison^21^), and reasoning (Raven Progressive Matrices;^23^ Educational Testing Service letter sets^24^). Additionally, we had a single measure of vocabulary (Educational Testing Service Vocabulary^24^). Baseline scores for each task were converted to z-scores in the 40- to 89-year-olds, and the follow-up scores were z-transformed using the baseline mean and standard deviation. Finally, raw MMSE scores were used as an estimate of overall cognitive status.

### MRI Protocol

Participants were scanned using a 3-T Philips Achieva scanner with an 8-channel head coil. High-resolution anatomical images were collected with a T1-weighted magnetization-prepared rapid gradient-echo sequence with 160 sagittal slices (field of view, 204 × 256 × 160 mm; voxel size, 1 × 1 × 1 mm^3^; time to repetition,  8.1 milliseconds; echo time, 3.7 milliseconds; flip-angle, 12°). Anatomical images were processed using FreeSurfer, version 5.3 (FreeSurfer) (http://surfer.nmr.mgh.harvard.edu/)^25^ with thorough manual editing.^27^ FreeSurfer volumetric segmentation was used to obtain cortical parcellations according to the Desikan-Killiany atlas.^28^

### PET Protocol

Participants were injected with a 370 MBq (10 mCi) bolus of ^18^F-florbetapir. A 2-frame by 5-minute each dynamic emission acquisition was started 50 minutes postinjection on the same ECAT HR PET scanner (Siemens Healthineers) for all participants. The detailed acquisition procedures were identical to those described previously.^6^ Each baseline PET scan was coregistered to the corresponding baseline MRI using FLIRT (https://fsl.fmrib.ox.ac.uk/fsl)^30^ with a mutual-information cost function. No partial volume correction was performed. The mean cortical SUVR was computed as a continuous measure of amyloid burden by averaging across 7 FreeSurfer-derived regions of interest (dorsolateral prefrontal, orbitofrontal, lateral parietal, lateral temporal, precuneus, isthmus cingulate, and rostral anterior cingulate cortices) and normalizing to the whole cerebellum.

Two dichotomous measures of amyloid status were generated. The first amyloid status variable defined positivity as in past studies,^13^ by setting the threshold at 2 SD above the mean SUVR for a young reference group (30- to 39-year-olds in our sample; SUVR threshold = 1.09). We also generated a second amyloid status variable using a more stringent threshold of 3 SD above the mean for the young reference group (SUVR threshold = 1.12). The resulting distributions across age can be found in eFigure 1 in the Supplement. Only participants aged 40 to 89 years were included in subsequent analyses.

### Data Analysis

For the primary analysis, linear mixed models were conducted with SUVR (treated as a continuous variable), the time of the test (baseline vs 4-year follow-up) and the SUVR × time interaction entered to predict changes in each cognitive measure. Baseline age, *APOE*, sex, and education were included as covariates. The covariate × time interactions were tested and removed if they did not approach significance (*P* > .10) to conserve statistical power. The age × SUVR × time interaction was tested to ensure that the effect of amyloid on cognitive decline did not differ as a function of age and did not approach significance in any model. Finally, cognitive score intercept was included as a random effect to account for individual differences in baseline performance. We also developed a less sensitive, but roughly analogous, nonparametric model by calculating Spearman correlations between SUVR and cognitive change scores while controlling for age, *APOE*, sex, education, and baseline cognitive performance.

A second linear mixed-model analysis identical to the previously described model was performed, but it included dichotomized amyloid status as a covariate, allowing us to assess whether continuous SUVR explained additional variance beyond dichotomized amyloid status. Next, we conducted the same linear mixed models analyses, removing continuous SUVR as a predictor, and examined the effect of dichotomized amyloid status on cognitive decline. Finally, subsample analyses were conducted separately on amyloid-negative and positive subgroups and on middle-aged adults (aged 40-59 years) and older adults (aged 60-89 years). All analyses were performed in SPSS, version 23 (IBM).

## Results

### Demographics

Table 1 presents descriptive information about the sample. Independent *t* tests indicated that amyloid-positive participants were older and more educated than amyloid-negative participants, and χ^2^ tests showed a trend for a higher proportion of carriers of *APOE ε4* among amyloid-positive participants. No demographic differences occurred when paired-samples *t* tests compared amyloid-positive and negative groups for the 2 thresholds (see eTable 1 in the Supplement for additional sample information).

**Table 1.** Sample Demographics^a^

	Whole Sample, Age 40-89 y (n = 174)	Amyloid Status, 2 SD		Amyloid Status, 3 SD	
		Amyloid-Positive (n = 49)	Amyloid-Negative (n = 125)	Amyloid-Positive (n = 31)	Amyloid-Negative (n = 143)
Age, mean (SD), y	66.44 (11.74)	71.98 (9.52)^b^	64.13 (11.77)^b^	72.86 (9.17)^b^	64.92 (11.74)^b^
SUVR, mean (SD)	1.09 (0.16)	1.27 (0.21)^b^	1.02 (0.03)^b^	1.37 (0.20)^b^	1.02 (0.03)^b^
Education, mean (SD), y	15.55 (2.29)	16.17 (2.49)^b^	15.29 (2.17)^b^	16.65 (2.15)^b^	15.30 (2.26)^b^
Time between visits, mean (SD), y	3.82 (0.32)	3.80 (0.31)	3.82 (0.33)	3.84 (0.23)	3.81 (0.34)
Men, No. (%)	65 (37)	48 (38)	16 (33)	55 (38)	9 (29)
*APOE ε4* carriers, No. (%)	38 (23)	14 (29)	24 (20)	11 (31)	27 (20)

Abbreviations: *APOE*, apolipoprotein; SUVR, standardized uptake value ratio.

^a^ Means (SD) (or percentages for categorical variables) are presented for all predictors and covariates for the full sample. Independent *t* tests indicated that amyloid-positive participants were older and more educated than amyloid-negative participants (at both thresholds) and χ^2^ tests showed a trend for a higher proportion of carriers of *APOE ε4* among amyloid-positive participants only for the 3-SD threshold. No demographic differences occurred when paired-samples *t* tests compared amyloid-positive and negative groups for the 2 thresholds.

^b^ *P* < .05.

### Dose-Response Relationship Between Amyloid and Cognitive Decline

#### Whole Sample (Ages 40-89 Years)

**Figure 1.**
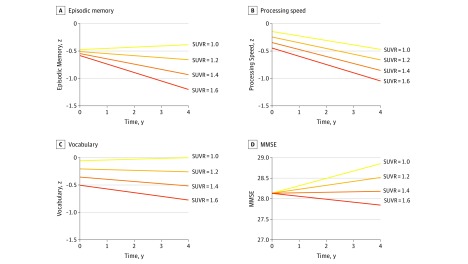
Projections of the Effect of Increasing Magnitude of Baseline Standardized Uptake Value Ratio (SUVR) Over 4 Years on 4 Measures of Cognition Linear mixed models were used to assess the effect of increasing baseline SUVR on the trajectory of cognitive performance from year 0 to year 4. The SUVR × time interaction was significant for episodic memory (EM) (A), processing speed (B), vocabulary (Vocab) (C), and Mini-Mental State Examination score (MMSE) (D), indicating that a dose-response relationship existed between baseline amyloid burden and cognitive change. The models consistently projected that increasing baseline values of SUVR were associated with increasing cognitive decline from year 0 to year 4 (see eMethods in the Supplement for description of the simple slope method used to generate projections). For episodic memory and vocabulary, estimated change at an SUVR of 1.0 reflected no change (EM = 0.09; Vocab = 0.06), while an SUVR of 1.6 was associated with declines (EM = −0.62; Vocab = −0.27). Declines in processing speed were predicted even at an SUVR of 1.0 (−0.33) but the rate of decline increased with SUVR such that an SUVR of 1.6 was associated with greater decline (−0.60). At an SUVR of 1.0, MMSE was associated with an increase (0.72), but this practice effect diminished with increasing SUVR and at an SUVR of 1.6 MMSE declined (−0.29).

The primary analysis examined the dose-response relationship between continuous baseline SUVR and cognitive change over 4 years. The analysis yielded significant SUVR × time interactions for 4 cognitive measures: episodic memory, processing speed, vocabulary, and MMSE, but not reasoning (Table 2). To interpret these interactions, simple slope analyses were used to project trajectories of cognitive change for 4 values of SUVR (1.0, 1.2, 1.4, and 1.6), holding all other fixed effects constant (Figure 1). These values were chosen as meaningful markers of the magnitude of amyloid burden, with 1.0 corresponding to amyloid negativity, 1.2 to low amyloid burden, 1.4 to moderate amyloid burden, and 1.6 to high burden. As shown in Figure 1, the 4 interactions occurred because increasing baseline SUVR was associated with increasing cognitive decline. A nonparametric Spearman correlation analysis yielded a significant correlation between changes in SUVR and MMSE (*r* = −0.168; 95% CI, −0.33 to −0.1; *P* = .03), a trend for episodic memory change (*r* =  −0.135; 95% CI, −.028 to 0.03; *P* = .08) and nonsignificant results for vocabulary (*r*, −0.099; 95% CI, −0.25 to 0.06; *P* = .20) and processing speed (*r*, −0.023; 95% CI, −0.19 to 0.14; *P* = .76), with confidence intervals based on 1000 bootstrap samples.

**Table 2.** Summary of Parameter Estimates From Linear Mixed Models for the Whole Sample (40- to 89-Year-Olds)^a^

Cognitive Outcome^b^	Episodic Memory (z)		Processing Speed (z)		Vocabulary (z)		Reasoning (z)		MMSE	
	Est (SE)	*P *Value	Est (SE)	*P *Value	Est (SE)	*P* Value	Est (SE)	*P* Value	Est (SE)	*P *Value
**Effects of Time**										
Time	1.27 (0.40)^b^	.002^b^	0.83 (0.27)^b^	.003^b^	0.60 (0.21)^b^	.004^b^	0.86 (0.37)^b^	.02^b^	0.87 (1.04)	.40
SUVR × time	−1.18 (0.37)^b^	.001^b^	−0.46 (0.22)^b^	.04^b^	−0.54 (0.19)^b^	.004^b^	−0.26 (0.30)	.39	−1.67 (0.75)^b^	.03^b^
Age × Time			−0.01 (0.00)^b^	.001^b^			−0.01 (0.00)^b^	.02^b^		
Ed. × time									0.10 (0.05)	.07
**Other Main Effects**										
SUVR	−0.18 (0.36)	.62	−0.50 (0.40)	.21	−0.75 (0.46)	.10	−0.74 (0.40)	.07	−0.01 (0.62)	.99
Age	−0.02 (0.00)^b^	<.001^b^	−0.04 (0.01)^b^	<.001^b^	0.02 (0.01)^b^	.004^b^	−0.03 (0.01)^b^	<.001^b^	−0.02 (0.01)^b^	.001^b^
Ed.	0.07 (0.02)^b^	.001^b^	0.04 (0.03)	.10	0.21 (0.03)^b^	<.001^b^	0.10 (0.03)^b^	<.001^b^	0.04 (0.04)	.44
Sex	0.76 (0.10)^b^	<.001^b^	0.38 (0.12)^b^	.002^b^	0.23 (0.14)	.10	0.06 (0.12)	.60	0.50 (.15)^b^	<.001^b^
* APOE*	0.003 (0.11)	.98	−0.21 (0.14)	.12	−0.03 (0.16)	.85	0.16 (0.13)	.22	−0.30 (0.18)	.10

Abbreviations: *APOE*, apolipoprotein; Ed, education; Est, estimate; MMSE, Mini-Mental State Examination; SUVR, standardized uptake value ratio.

^a^ Parameter estimates, standard error, and *P* values are reported above for each cognitive outcome. The primary predictor of interest, the SUVR × time interaction, was significant for episodic memory, processing speed, vocabulary, and MMSE, indicating a significant dose-response relationship between baseline amyloid burden and cognitive change. There was also a significant positive main effect of time for episodic memory, processing speed, vocabulary, and reasoning, indicating that there was an increase over time in these variables independent of amyloid burden. Next, we detected a significant age × time interaction for processing speed and reasoning, such that old age was also associated greater cognitive decline, independent of amyloid burden. The age × time interaction failed to reach marginal significance for the remaining cognitive variables and was removed from the models (eFigure 2 in the Supplement). There was a marginally significant education × time interaction, such that increasing education was associated with more positive change in MMSE. The education × time interaction failed to reach marginal significance for the other cognitive variables and was removed from the models. *APOE* × time, sex × time, and age × SUVR × time estimates for all cognitive variables failed to reach marginal significance and were removed from the models.

^b^ *P* < .05.

**Figure 2.**
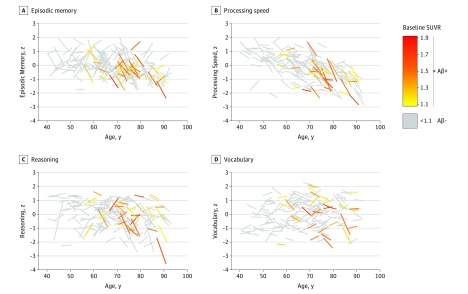
Cognitive Change in Individual Participants Over 4 Years as a Function of Age Gray lines represent amyloid-negative participants in terms of episodic memory (A), processing speed (B), reasoning (C), and vocabulary (D). Amyloid-positive individuals are shown in color, with the color scale ranging from yellow (lowest standardized uptake value ratio [SUVR]) to red (highest SUVR).

Figure 2 depicts individual trajectories of change for participants as a function of age and amyloid burden. Although there was considerable variability in individual trajectories of cognitive change, declines were more consistently observed in older adults with high amyloid burden.

Next, we examined the SUVR × time interaction, while controlling for dichotomized amyloid status. Using the 2-SD amyloid status variable as a covariate, the interaction remained significant for episodic memory (Estimate [Est] [SE] = −1.20 [0.52]; 95% CI, −2.22 to 0.18; *P* = .02), vocabulary (Est [SE] = −0.54 [0.26]; 95% CI, −1.06 to −0.02; *P* = .04) and MMSE (Est [SE] = −2.17 [1.05]; 95% CI, −4.24 to −0.11; *P* = .04). Using the more stringent 3-SD threshold, the SUVR × time interaction remained significant for MMSE (Est [SE]* =* −2.54 [1.27]; 95% CI, −5.05 to −0.03; *P* = .05*)* and marginally significant for episodic memory (Est [SE]* =* −1.18 [0.63]; 95% CI, −2.42 to 0.07; *P* = .06*)* (eTable 2 in the Supplement).

#### Dichotomized Amyloid Status

**Figure 3.**
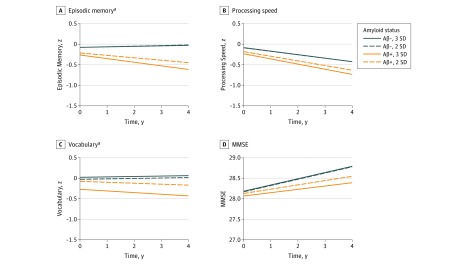
Projections of the Effect of Amyloid Positivity Over 4 Years on 4 Measures of Cognition Lines represent trajectories of change between estimated marginal means of cognitive performance at year 0 and year 4 for the amyloid-positive and negative groups, at both 2-SD and 3-SD thresholds. Cognitive decline significantly differed as a function of amyloid status for episodic memory (EM) (A) and vocabulary (Vocab) (C), but not processing speed (B) or Mini-Mental State Examination (MMSE) (D) at both 2-SD and 3-SD thresholds. Amyloid-positive individuals exhibited modest declines in episodic memory (2-SD EM,−0.24; 3-SD EM, −0.35) and vocabulary (2-SD Vocab,−0.09; 3-SD Vocab, −0.16), while the amyloid-negative individuals did not change in episodic memory (2-SD EM, 0.06; 3-SD EM, 0.05) or vocabulary (2-SD Vocab, 0.04; 3 SD Vocab, 0.04). By contrast, the continuous standardized uptake value ratio (SUVR) analyses (Figure 1) yielded a dose-response effect of amyloid on declines in all 4 of these variables, and provided more detailed information about the increasing trajectory of declines at different SUVR values. ^a^*P* < .05.

We also modeled the effect of dichotomized amyloid status on cognitive decline. Using the 2-SD amyloid status variable, we found significant amyloid status × time interactions for episodic memory (Est [SE] = −0.29 [0.13]; 95% CI, −0.55 to −0.03; *P* = *.*03) and vocabulary (Est [SE] *=* −0.14 [0.07]; 95% CI, −0.27 to −0.004; *P* = .04), while processing speed (Est [SE] = −0.11 [0.08]; 95% CI, −0.27 to 0.05; *P* = .16) and MMSE (Est [SE] = -0.19 [0.27]; 95% CI, −0.73 to 0.35; *P* = .47) were not significant. Using the amyloid at 3 SD variable, we again found significant amyloid status × time interactions for only episodic memory (Est [SE] = −0.40 [0.15]; 95% CI, −0.70 to −0.10;* P =* .01) and vocabulary (Est [SE]* *= −0.20 [0.08]; 95% CI, −0.35 to −0.05;* P =* .01) (eTable 3 in the Supplement). Figure 3 shows that at both thresholds, amyloid-positive participants exhibited decline in episodic memory and vocabulary while amyloid-negative participants did not.

#### Amyloid-positive and Negative Subsample Analyses

To further verify the continuous relationship of amyloid burden to cognitive decline, we conducted the same dose-response analyses separately for amyloid-positive and negative participants. For the amyloid-positive group using the 2-SD threshold (n = 49), the SUVR × time interaction was again significant for episodic memory (Est [SE] = −1.33 [0.54]; *P* = .02), and approached significance for MMSE (Est [SE] = −1.91 [1.05]; *P* = .08) (eTable 4 and eFigure 3 in the Supplement). Using the more stringent 3-SD threshold (n = 31), the SUVR × time interaction was significant only for episodic memory (Est [SE] = −1.36 [0.66]; 95% CI, −2.71 to −0.01; *P* = .05). For the amyloid-negative group, significant SUVR × time interactions were not detected for any cognitive measure at either threshold (eTable 5 in the Supplement).

#### Middle-aged and Older Adult Subsample Analyses

Although amyloid burden was lower among middle-aged adults compared with older adults (Figure 2; eFigure 1 in the Supplement), we considered that a dose-relationship could still exist among only middle-aged adults (n = 51). The analyses yielded 1 significant SUVR × time interaction for vocabulary (Est [SE] = −2.05 [0.86]; 95% CI, −3.78 to −0.33; *P* = .02) (eTable 6 in the Supplement). However, the interaction was driven by 1 individual with the highest SUVR and greatest vocabulary decline (eFigure 4 in the Supplement). When removed, the interaction became nonsignificant (*P* = .21). Interestingly, post hoc examinations revealed that this outlier and the 2 other individuals with the highest SUVR in middle age were *APOE ε4*/*ε4* and exhibited vocabulary decline.

Finally, for a comparison with other studies that included only older adults, we repeated the analyses among 60- to 89-year-olds only. Like the whole sample, we found significant SUVR × time interactions for episodic memory (Est [SE] = −1.03 [0.38]; 95% CI, −1.79 to −0.29; *P* = .01), processing speed (Est [SE] = −0.53 [0.22]; 95% CI, −0.97 to −0.09;* P =* .02), vocabulary (Est [SE]* = −*0.46 [0.19]; 95% CI, −0.84 to −0.08;* P =* .02) and MMSE (Est [SE] = -1.63 [0.79]; 95% CI, −3.19 to −0.05; *P* = .04) (eTable 7 and eFigure 5 in the Supplement).

## Discussion

This study provides evidence of a dose-response effect in which the magnitude of baseline amyloid burden predicted the rate of cognitive decline over 4 years in cognitively normal adults, even after controlling for dichotomized amyloid status. These results suggest that the degree of amyloid burden provides potentially important additional information about the rate of expected cognitive decline that is not available from a dichotomous positive/negative categorization. These findings may have important implications for projecting clinical outcomes on the basis of an amyloid PET scan, as well as for understanding the effect of amyloid in preclinical AD. Additionally, we report the effects of amyloid on cognitive decline among middle-aged adults, who to our knowledge have rarely been studied, and found limited evidence for a dose-response relationship in this group.

### Dose-Response Effect of Baseline Amyloid on Cognitive Decline

Episodic memory decline is the signature behavioral characteristic of AD, and longitudinal studies that model amyloid as a dichotomous variable have confirmed that the presence of amyloid in cognitively normal adults predicts greater episodic memory decline.^12^ Here, we observed a dose-response effect of baseline amyloid burden on 4-year episodic memory decline. At low SUVR, there was no change in episodic memory, but increasing SUVR predicted steeper rates of episodic memory decline. These findings remained after controlling for amyloid positivity as well as when the analysis sample was limited to only amyloid-positive adults. In addition to episodic memory, we also found significant dose-response effects across several cognitive variables (processing speed, vocabulary, and MMSE) that attested to the broad effect of amyloid.

In a related study, Lim et al^16^ focused on amyloid-positive adults, combining cognitively normal participants with those with mild cognitive impairment and dichotomizing them into low and high amyloid groups. They reported a greater memory decline for the group with higher amyloid compared with the group with lower amyloid. This study expands on these findings by demonstrating a continuous dose-response relationship between amyloid burden and cognitive decline across a range of domains in healthy adults. These findings suggest that the magnitude of amyloid burden may be useful in projecting the rate of future cognitive decline, with those with the greatest burdens showing the most decline. Similarly, Chételat et al^35^ demonstrated a dose-response effect of amyloid burden on cortical atrophy, suggesting that a dose-response effect may generalize to other AD biomarkers.

### Dichotomous vs Continuous Amyloid

When we treated amyloid as a dichotomous variable, analyses yielded fewer significant effects of amyloid on cognitive decline, and the decline trajectories appeared to be more modest for amyloid status (Figure 3) compared with the results for continuous SUVR (Figure 1). Furthermore, when dichotomized amyloid status was included as a covariate, the continuous SUVR effect remained significant for several cognitive domains. This provides additional evidence supporting using a dose-response approach.

We also found that varying the positivity threshold used to define dichotomized amyloid status resulted in somewhat different outcomes while predicting cognitive decline. Significant effects of amyloid status alone on cognitive decline were observed for episodic memory and vocabulary using both thresholds, although the effects using a threshold 3-SDs above the young adult mean were of slightly greater magnitude than the effects using the more liberal 2-SD threshold. However, when amyloid status was included as a covariate in the continuous SUVR analysis, the dose-response effect remained significant for episodic memory, vocabulary and MMSE using the 2-SD threshold, but was significant only for MMSE when using the 3-SD threshold. These findings highlight that dichotomous measures of amyloid may omit useful information and that selecting a positivity threshold can influence results. Our results, when combined with previous findings,^16^ may have implications for the recently proposed amyloid/tau/neurodegeneration framework^36^ that relies on the dichotomous classification of multiple AD biomarkers.

### Amyloid and Middle Age

Although the SUVR uptake observed in middle age is typically relatively low,^6^ we hypothesized that middle-aged individuals with a high SUVR relative to their peers would exhibit steeper cognitive declines. In this group, there was a dose-response relationship of amyloid burden on vocabulary decline only. We noted, post hoc, that this result was driven by 3 outliers who had the highest SUVRs and who on closer examination were found to be the only 3 participants with the *APOE ε4/ε4* allele in the 40- to 59-year age group. This finding, while anecdotal, provides qualitative evidence suggesting that *APOE ε4* is an important factor in preclinical AD in middle age. This is consistent with autopsy findings^37^ that demonstrate that amyloid plaques are already prevalent in middle age (particularly among 50- to 59-year olds) in carriers of *APOE ε4*.

Recent findings^38^ provided some impetus for the possibility that relatively high amyloid burden within the subthreshold range may be predictive of future cognitive decline. However, we failed to find evidence for this in our results when we treated SUVR as a continuous variable among the amyloid-negative participants or in middle age after removing the *APOE ε4/ε4s* homozygotes. It is possible, however, that nonspecific binding in the amyloid-negative range obscured the effect of low amyloid deposition, especially as no corrections for partial volume effects were performed. Longer follow-ups and longitudinal measurements of increasing amyloid burdens may allow for the detection of subthreshold effects of amyloid on cognition.

### Limitations

It is possible that this sample may be underpowered to detect subtle effects, particularly in the subsamples. We also note that the effects were more limited when less powerful nonparametric analyses were applied to accommodate the non-normal distribution that is characteristic of amyloid. However, the amyloid-positive sample has a more normal distribution, and the dose-response effect remains significant for episodic memory among amyloid-positive participants. Finally, because our sample was recruited based on being healthy, we do not have assessments of clinical function on these participants, which would be desirable as the participants age and potentially progress to developing mild cognitive impairment or AD. However, these findings establish that increasing amyloid burden is predictive of cognitive decline in our sample, regardless of clinical status.

## Conclusions

We demonstrate a dose-response relationship between the magnitude of amyloid burden at baseline and the rate of cognitive decline over a 4-year follow-up in healthy adults, particularly for episodic memory. These results suggest that the magnitude of amyloid deposition predicts those likely to be on a more negative cognitive trajectory, potentially heading toward dementia, and provides potentially important additional information about the rate of expected cognitive decline that is not available from a dichotomous positive/negative categorization.

## Notes

Supplement. **eMethods.** Description of Simple Slope Method for SUVR x Time Figures. **eFigure 1. **Amyloid Burden Throughout the Lifespan. **eFigure 2. **Increasing Age at Baseline is Associated With Increasing Decline in Processing Speed and Reasoning in Amyloid-negative Adults. **eFigure 3. **Dose-response Relationship Between Baseline Amyloid Burden and Episodic Memory Decline in Amyloid-Positive Individuals. **eFigure 4. **Dose-Response Relationship Between Baseline Amyloid Burden and Vocabulary in Middle-Aged Adults Driven by Three APOE-ε4 Homozygotes. **eFigure 5. **Projections of the Impact of Increasing Magnitude of Baseline SUVR over a 4-Year Time Interval on 4 Measures of Cognition in Older Adults. **eTable 1**. Sample Demographics. **eTable 2.** Summary of Parameter Estimates from Linear Mixed Models With Both Amyloid Status and SUVR. **eTable 3.** Summary of Parameter Estimates from Linear Mixed Models With Amyloid Status Instead of SUVR. **eTable 4. **Summary of Parameter Estimates from Linear Mixed Models for Amyloid-Positive Adults Only. **eTable 5.** Summary of Parameter Estimates from Linear Mixed Models for Amyloid-Negative Adults Only. **eTable 6.** Summary of Parameter Estimates from Linear Mixed Models for Middle-Aged Adults Only. **eTable 7**. Summary of Parameter Estimates from Linear Mixed Models for Older Adults Only. **eTable 3.** Summary of Parameter Estimates from Linear Mixed Models With Amyloid Status Instead of SUVR.
