# Supplementary material for: Association of Longitudinal Cognitive Decline With Amyloid Burden in Middle-aged and Older Adults: Evidence for a Dose-Response Relationship
Source: JAMA Neurol. 2017 Jul 10;74(7):830–8. doi: 10.1001/jamaneurol.2017.0892 (PMC5710531; doi:10.1001/jamaneurol.2017.0892)
Supplement: Supplement. — eMethods. Description of Simple Slope Method for SUVR x Time Figures. eFigure 1. Amyloid Burden Throughout the Lifespan. eFigure 2. Increasing Age at Baseline is Associated With Increasing Decline in Processing Speed and Reasoning in Amyloid-negative Adults. eFigure 3. Dose-response Relationship Between Baseline Amyloid Burden and Episodic Memory Decline in Amyloid-Positive Individuals. eFigure 4. Dose-Response Relationship Between Baseline Amyloid Burden and Vocabulary in Middle-Aged Adults Driven by Three APOE-ε4 Homozygotes. eFigure 5. Projections of the Impact of Increasing Magnitude of Baseline SUVR over a 4-Year Time Interval on 4 Measures of Cognition in Older Adults. eTable 1. Sample Demographics. eTable 2. Summary of Parameter Estimates from Linear Mixed Models With Both Amyloid Status and SUVR. eTable 3. Summary of Parameter Estimates from Linear Mixed Models With Amyloid Status Instead of SUVR. eTable 4. Summary of Parameter Estimates from Linear Mixed Models for Amyloid-Positive Adults Only. eTable 5. Summary of Parameter Estimates from Linear Mixed Models for Amyloid-Negative Adults Only. eTable 6. Summary of Parameter Estimates from Linear Mixed Models for Middle-Aged Adults Only. eTable 7. Summary of Parameter Estimates from Linear Mixed Models for Older Adults Only. eTable 3. Summary of Parameter Estimates from Linear Mixed Models With Amyloid Status Instead of SUVR. [file jamaneurol-74-830-s001.pdf]

Supplementary Online Content

Farrell ME, Kennedy KM, Rodrique KM, et al. Association of longitudinal cognitive decline with amyloid burden in middle-aged and older adults: evidence for a dose-response relationship. *JAMA Neurol*. Published online May 30, 2017. doi:10.1001/jamaneurol.2017.0892

eMethods. Description of Simple Slope Method for SUVR x Time Figures
eFigure 1. Amyloid Burden throughout the Lifespan
eFigure 2. Increasing Age at Baseline is Associated with Increasing Decline in Processing Speed and Reasoning in Amyloid Negative Adults
eFigure 3. Dose-response Relationship Between Baseline Amyloid Burden and Episodic Memory Decline in Amyloid-Positive Individuals
eFigure 4. Dose-Response Relationship Between Baseline Amyloid Burden and Vocabulary in Middle-Aged Adults Driven by Three APOE-ε4 Homozygotes
eFigure 5. Projections of the Impact of Increasing Magnitude of Baseline SUVR over a 4-Year Time Interval on 4 Measures of Cognition in Older Adults
eTable 1. Sample Demographics
eTable 2. Summary of Parameter Estimates from Linear Mixed Models With Both Amyloid Status and SUVR
eTable 3. Summary of Parameter Estimates from Linear Mixed Models With Amyloid Status Instead of SUVR
eTable 4. Summary of Parameter Estimates from Linear Mixed Models for Amyloid-Positive Adults Only
eTable 5. Summary of Parameter Estimates from Linear Mixed Models for Amyloid-Negative Adults Only
eTable 6. Summary of Parameter Estimates from Linear Mixed Models for Middle-Aged Adults Only
eTable 7. Summary of Parameter Estimates from Linear Mixed Models for Older Adults Only

This supplementary material has been provided by the authors to give readers additional information about their work.

© 2017 Farrell ME et al. *JAMA Neurol.*

eMethods. Description of Simple Slope Method for SUVR x Time Figures

In order to evaluate the cause of the significant SUVR x time interactions, estimated means were computed for four values of SUVR that ranged from low to high (1.0, 1.2, 1.4, 1.6) across four years for each cognitive measure that was significant. Estimated means were computed by first building the full linear model for each cognitive variable with all fixed effects and their associated parameter estimates as generated by the linear mixed model. Next, the x value of each term was entered into the linear model. Covariate terms in the model were set to defaults for categorical variables (Sex=male; APOE: non-carrier) and means for continuous variables (Age=66.4, Education years =15.6). Time was set to 0 to generate baseline predicted values and 1 to generate follow-up values. Finally, each of the 5 values of SUVR was entered separately to generate model projections of cognitive performance at baseline and at the follow-up interval four years later. The baseline and follow-up model projections for each of the five SUVR values were then used to estimate trajectories of change in cognition over time at increasing values of SUVR.

© 2017 Farrell ME et al. *JAMA Neurol.*

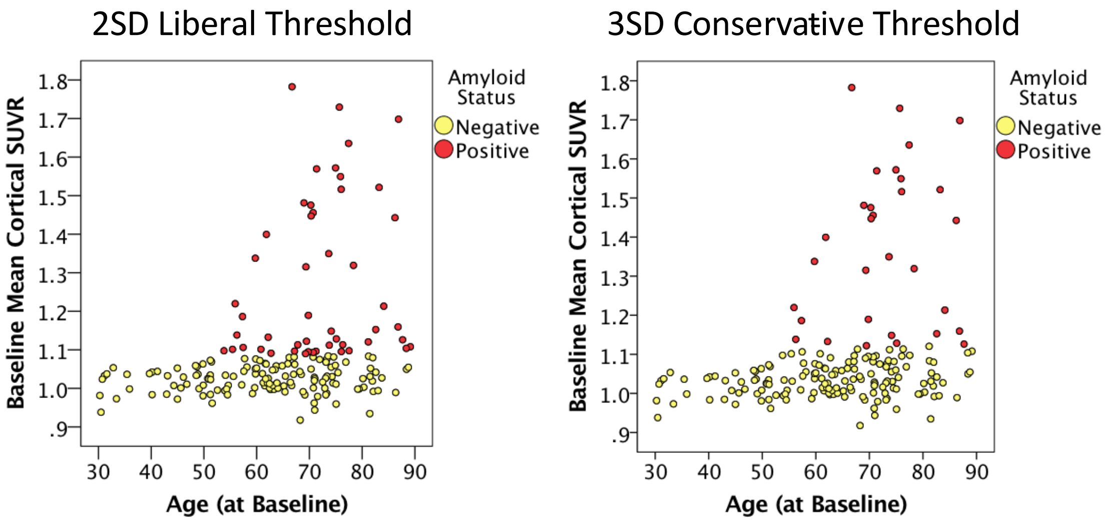

eFigure 1. Amyloid Burden throughout the Lifespan with Different Positivity Thresholds.
Amyloid burden at baseline (Baseline Mean Cortical SUVR) is plotted as a function of age at baseline. Additionally, the dichotomization of SUVR into Amyloid Positive (red) and Amyloid Negative (yellow) groups is shown, with a threshold at 1.09 (left) and 1.12 (right) based on 2 or 3 standard deviations, respectively, above the mean uptake in the youngest subjects (age 30-39).

© 2017 Farrell ME et al. *JAMA Neurol.*

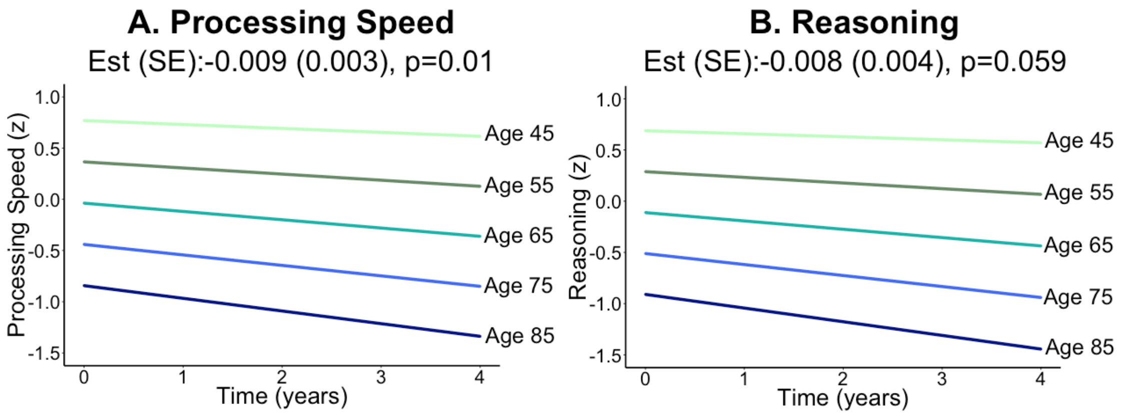

eFigure 2. Increasing Age at Baseline associated with Increasing Decline in Processing Speed and Reasoning in Amyloid Negative Adults. Using simple slope analysis, the projected trajectory of (A) processing speed and (B) reasoning change over time was plotted for different ages in the amyloid-negative subsample. Increasing age was predictive of greater decline in processing speed, and marginally predictive of greater decline in reasoning

.

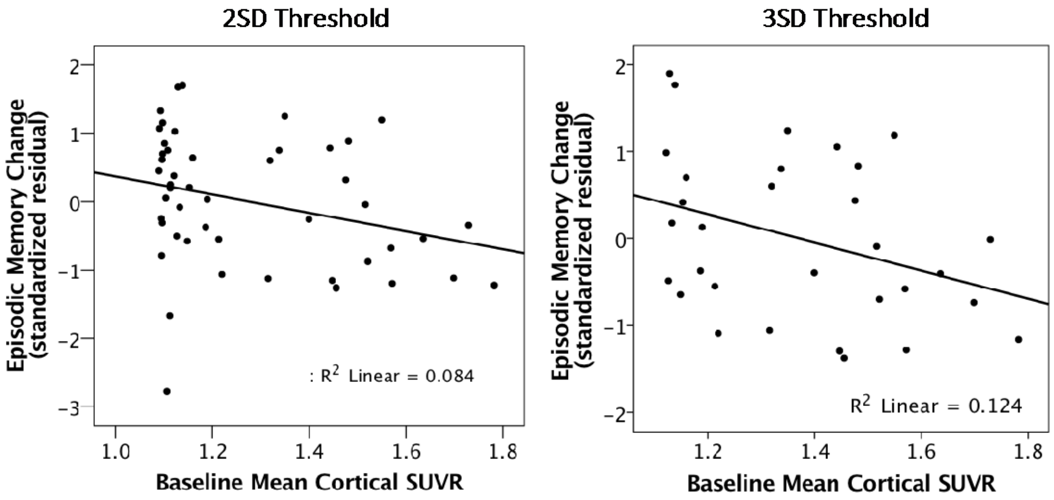

eFigure 3. Dose-Response Relationship between Baseline Amyloid Burden and Episodic Memory Decline in Amyloid-Positive Individuals.
Scatterplots of the individual episodic memory change scores are shown (adjusted for age, sex, education and APOE) as a function of baseline SUVR for the amyloid positive subjects, based on the 2SD (left) and 3SD (right) thresholds. Within this smaller sample, increasing SUVR predicted increasingly negative trajectories of change in episodic memory.

© 2017 Farrell ME et al. *JAMA Neurol.*

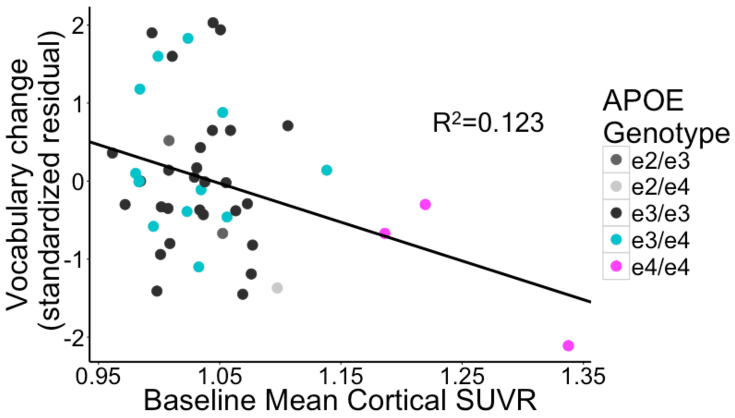

eFigure 4. Dose-Response Relationship Between Baseline Amyloid Burden and Vocabulary in Middle-Aged Adults Driven by Three APOE-ε4 Homozygotes.

Restricting the analysis to middle-aged adults (age 40-59 at baseline), yielded a significant SUVR x Time interaction for vocabulary with increasing SUVR predicting declining vocabulary. However, a post-hoc examination of the three participants with the highest SUVRs (shown in magenta) revealed that these three were APOE ε4/ε4 homozygotes—the only three in this middle-aged sample. When these three participants were removed from the analysis, the effect became non-significant.

© 2017 Farrell ME et al. *JAMA Neurol.*

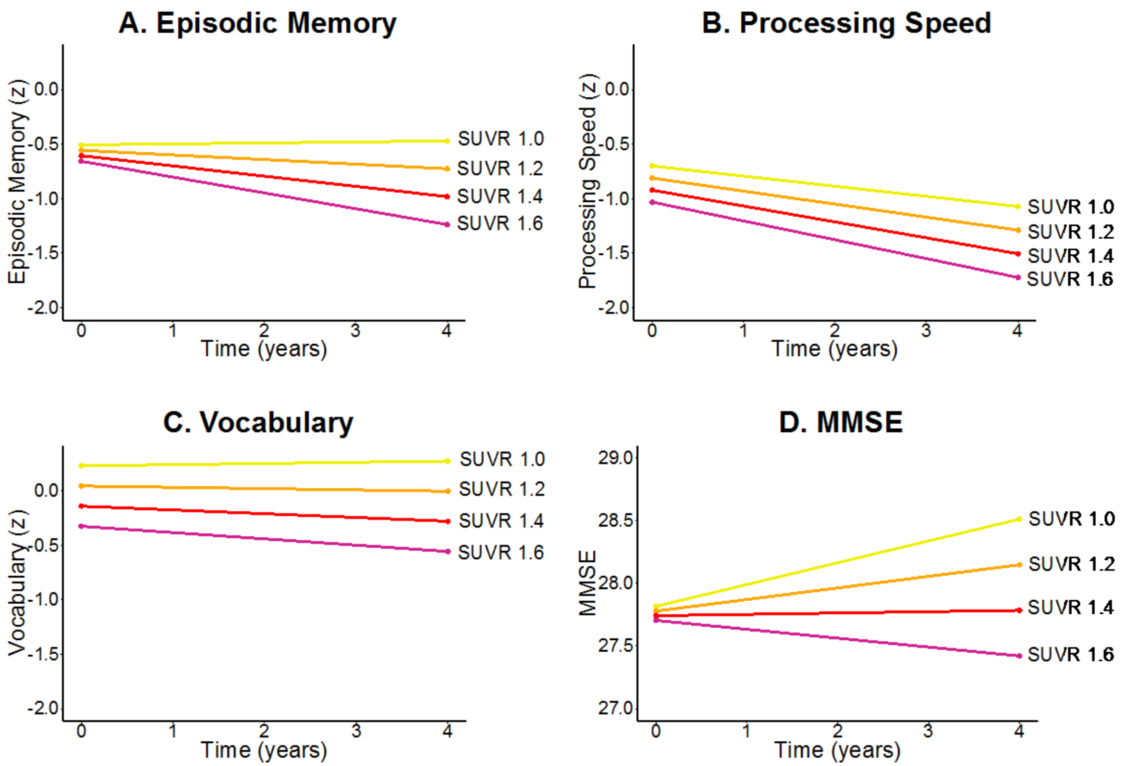

eFigure 5. Projections of the Impact of Increasing Magnitude of Baseline SUVR over a 4-Year Time Interval on 4 Measures of Cognition in Older Adults. Linear Mixed Models were used to assess the impact of increasing baseline amyloid burden (SUVR) on the trajectory of cognitive performance from Year 0 to Year 4 in older adults alone (age 60-89 at baseline). Similar to the results in the whole sample (age 40-89), the SUVR x Time interaction was significant for (a) episodic memory, (b) processing speed, (c) vocabulary and (d) MMSE, indicating a dose-response relationship between baseline amyloid burden and cognitive change. In order to evaluate the significant SUVR x time interactions, estimated means were computed for each time point based on increasing values of SUVR (1.0, 1.2, 1.4, 1.6). Across episodic memory, processing speed, vocabulary and MMSE, the model projected that high levels of SUVR (1.6) consistently predicted cognitive decline, while negative or low levels of SUVR (1.0, 1.2) predicted modest or no cognitive decline (or in the case of MMSE, a practice effect).

© 2017 Farrell ME et al. *JAMA Neurol.*

© 2017 Farrell ME et al. *JAMA Neurol.*

		Whole Sample (age 40-89) (*n*=174)	Baseline Amyloid Status: Amyloid Positive (n=31)	Amyloid Negative (n=143)	*t* (or *χ2*)	*p*
**Age (years)**		66.44 ± 11.74	*72.86 ± 9.18*	*65.06 ± 11.80*	*-3.46*	*0.001*[a]
**Baseline SUVR**		1.09 ± 0.16	*1.37 ± 0.20*	*1.04 ± 0.04*	*-18.44*	*<.001*
**Education (years)**		15.55 ± 2.29	*16.65 ± 2.15*	*15.32 ± 2.27*	*-2.98*	*0.003*
**Time between visits (years)**		3.82 ± 0.32	3.84 ± 0.23	3.81 ± 0.34	-0.43	0.667
**Gender (No. male (%))**		65 (37%)	9 (29%)	56 (39%)	1.06	0.303
**APOE (No. ε4 carrier (%))**		38 (23%)	11 (36%)	27 (20%)	3.68	0.055
**MMSE**	**Year 0**	28.3 ± 1.24	28.2 ± 1.21	28.3 ± 1.24	0.71	0.478
	**Year 4**	28.9 ± 1.31	28.5 ± 1.21	28.9 ± 1.32	1.76	0.080
	**Change**	0.55 ± 1.55	0.32 ± 1.74	0.60 ± 1.51	0.91	0.366
**Episodic Memory (*z*)**	**Year 0**	-0.01 ± 0.81	-0.14 ± 0.67	0.02 ± 0.84	0.98	0.331
	**Year 4**	-0.06 ± 0.85	*-0.49 ± 0.99*	*0.03 ± 0.81*	*3.17*	*0.002*
	**Change**	-0.05 ± 0.78	*-0.35 ± 0.77*	*0.01 ± 0.78*	*2.36*	*0.019*
**Processing Speed (*z*)**	**Year 0**	0.01 ± 0.95	*-0.34 ± 0.92*	*0.09 ± 0.94*	*2.32*	*0.021*
	**Year 4**	-0.36 ± 1.00	*-0.90 ± 0.99*	*-0.25 ± 0.97*	*3.41*	*0.001*
	**Change**	-0.37 ± 0.47	*-0.56 ± 0.53*	*-0.33 ± 0.44*	*2.52*	*0.013*
**Reasoning (*z*)**	**Year 0**	-0.00 ± 0.90	-0.29 ± 0.88	0.06 ± 0.90	1.92	0.056
	**Year 4**	-0.09 ± 0.97	*-0.55 ± 1.01*	*0.01 ± 0.94*	*2.93*	*0.004*
	**Change**	-0.09 ± 0.61	-0.26 ± 0.75	-0.05 ± 0.58	1.74	0.084
**Vocabulary (*z*)**	**Year 0**	0.00 ± 1.00	0.11 ± 1.15	-0.04 ± 0.96	-0.76	0.448
	**Year 4**	-0.01 ± 0.97	-0.05 ± 1.21	0.00 ± 0.92	0.27	0.787
	**Change**	0.01 ± 0.39	*-0.16 ± 0.48*	*0.04 ± 0.36*	*2.65*	*0.009*

eTable 1. Sample demographics.

© 2017 Farrell ME et al. *JAMA Neurol.*

Means and standard deviations (or percentages for categorical variables) are presented for all predictors, covariates and cognitive outcome variables for the full sample. Independent t-tests and chi-square tests were used to test for differences between amyloid positive and negative groups, using the more stringent 3SD threshold. Individuals who were amyloid positive at baseline were older, more educated, and marginally more likely to be APOE e4 carriers than amyloid negative individuals. Amyloid positive individuals also exhibited greater decline (based on change scores) in episodic memory, processing speed, vocabulary and a trend for reasoning than amyloid negative individuals. However, these change scores are unadjusted for covariates, and the primary analyses with linear mixed models are more appropriate tests to properly measure the relationship between amyloid burden and cognitive decline while accounting for possible confounds with covariates such as age. [a]$p<.05$ in italics

© 2017 Farrell ME et al. *JAMA Neurol.*

	Cognitive Outcome		Episodic Memory (z)		Processing Speed (z)		Vocabulary (z)		MMSE	
	**Effect**	**Statistic**	**3SD Aβ Status Threshold**	**2SD Aβ Status Threshold**	**3SD Aβ Status Threshold**	**2SD Aβ Status Threshold**	**3SD Aβ Status Threshold**	**2SD Aβ Status Threshold**	**3SD Aβ Status Threshold**	**2SD Aβ Status Threshold**
**Effects of Time**	**Time**	*Est (SE)*	1.26 (0.87)	**1.46 (0.70)**	0.74 (0.53)	0.82 (0.43)	0.45 (0.45)	0.60 (0.34)	2.25 (1.93)	1.65 (1.53)
		p	0.151	**0.042**	0.165	0.061	0.317	0.079	0.246	0.282
	**SUVR x Time**	*Est (SE)*	-1.18 (0.63)	**-1.20 (0.52)**	-0.41 (0.37)	-0.45 (0.30)	-0.44 (0.32)	**-0.54 (0.26)**	**-2.54 (1.27)**	**-2.17 (1.05)**
		p	0.064	**0.021**	0.271	0.133	0.170	**0.040**	**0.048**	**0.039**
	**Aβ Status x Time**	*Est (SE)*	0.00 (0.26)	-0.01 (0.18)	0.03 (0.15)	0.00 (0.11)	0.05 (0.13)	-0.00 (0.09)	-0.45 (0.53)	-0.26 (0.37)
		p	0.987	0.96	0.862	0.987	0.701	0.993	0.398	0.487
	**Age x Time**	*Est (SE)*	-	-	**-0.01 (0.00)**	**-0.01 (0.00)**	-			
		p			**0.001**	**0.001**				
**Other Main Effects**	**SUVR**	*Est (SE)*	0.46 (0.59)	0.33 (0.52)	-0.56 (0.64)	-0.57 (0.53)	-0.49 (0.73)	-1.12 (0.60)	0.35 (1.01)	0.05 (0.83)
		p	0.429	0.53	0.384	0.28	0.501	0.063	0.729	0.948
	**Aβ Status**	*Est (SE)*	0.34 (0.24)	0.18 (0.17)	-0.03 (0.27)	-0.04 (0.19)	0.14 (0.30)	-0.21 (0.21)	0.18 (0.42)	0.03 (0.30)
		p	0.158	0.29	0.911	0.839	0.651	0.334	0.66	0.932
	**Age**	*Est (SE)*	**-0.02 (0.00)**	**-0.02 (0.00)**	**-0.04 (0.01)**	**-0.04 (0.01)**	**0.02 (0.01)**	**0.02 (0.01)**	**-0.02 (0.01)**	**-0.02 (0.01)**
		p	**<0.001**	**<0.001**	**<0.001**	**<0.001**	**0.003**	**0.007**	**0.001**	**0.001**
	**Ed.**	*Est (SE)*	**0.08 (0.02)**	0.08 (0.05)	0.04 (0.03)	0.04 (0.03)	**0.21 (0.03)**	**0.21 (0.03)**	0.04 (0.04)	0.03 (0.04)
		p	**<0.001**	0.108	0.102	0.104	**<0.001**	**<0.001**	0.387	0.413
	**Sex**	*Est (SE)*	**0.78 (0.10)**	**0.86 (0.21)**	**0.38 (0.12)**	**0.38 (0.12)**	0.24 (0.14)	0.22 (0.14)	**0.50 (0.15)**	**0.50 (0.15)**
		p	**<0.001**	**<0.001**	**0.002**	**0.002**	0.087	0.12	**0.001**	**0.002**
	**APOE**	*Est (SE)*	-0.01 (0.11)	0.01 (0.11)	0.21 (0.14)	-0.22 (0.14)	0.02 (0.16)	-0.03 (0.16)	0.30 (0.18)	-0.30 (0.18)
		p	0.893	0.955	0.123	0.122	0.88	0.829	0.095	0.093

eTable 2. Summary of Parameter Estimates from Linear Mixed Models with both Amyloid Status and SUVR.
Parameter estimates and standard errors are reported above for each cognitive outcome in the whole sample. Both SUVR and Amyloid Status (2SD or 3SD threshold) was included in these models, to assess whether continuous SUVR was a significant predictor of cognitive decline even

© 2017 Farrell ME et al. *JAMA Neurol.*

after accounting for amyloid status. Even while accounting for Amyloid Status (2SD threshold), the Amyloid Status x Time interaction was significant for episodic memory, vocabulary, and MMSE. Accounting for Amyloid Status (3SD threshold), MMSE remained significant and episodic memory was marginally significant. $p<.05$ in bold.

© 2017 Farrell ME et al. *JAMA Neurol.*

	Cognitive Outcome		Episodic Memory (z)		Processing Speed (z)		Vocabulary (z)		Reasoning (z)		MMSE	
	**Effect**	**Statistic**	**2SD Aβ Status**	**3SD Aβ Status**	**2SD Aβ Status**	**3SD Aβ Status**	**2SD Aβ Status**	**3SD Aβ Status**	**2SD Aβ Status**	**3SD Aβ Status**	**2SD Aβ Status**	**3SD Aβ Status**
**Effects of Time**	**Time**	*Est (SE)*	0.35 (0.18)	**-0.35 (0.14)**	**0.49 (0.20)**	0.22 (0.24)	0.18 (0.09)	**-0.16 (0.07)**	**0.68 (0.28)**	0.47 (0.33)	**0.80 (0.36)**	0.32 (0.28)
		p	0.053	**0.011**	**0.017**	0.363	0.053	**0.023**	**0.016**	0.151	**0.029**	0.250
	**Aβ Status x Time**	*Est (SE)*	**-0.29 (0.13)**	**-0.40 (0.15)**	-0.11 (0.08)	-0.16 (0.09)	**-0.14 (0.07)**	**-0.20 (0.08)**	-0.09 (0.11)	-0.12 (0.13)	-0.19 (0.27)	-0.28 (0.31)
		p	**0.028**	**0.009**	0.161	0.081	**0.044**	**0.011**	0.412	0.327	0.472	0.361
	**Age x Time**	*Est (SE)*		-	**-0.01 (0.00)**	-0.01 (0.00)		-	**-0.01 (0.00)**	**-0.01 (0.00)**		
		p			**0.001**	0.001			**.022**	**0.019**		
**Other Main Effects**	**Aβ Status**	*Est (SE)*	0.14 (0.13)	-0.19 (0.15)	-0.09 (0.14)	-0.16 (0.17)	-0.05 (0.16)	-0.29 (0.19)	-0.18 (0.14)	-0.28 (0.17)	-0.04 (0.22)	-0.01 (0.26)
		p	0.292	0.210	0.524	0.369	0.772	0.123	0.222	0.099	0.856	0.651
	**Age**	*Est (SE)*	**-0.02 (0.00)**	-0.02 (0.00)	**-0.04 (0.01)**	-0.04 (0.01)	**0.02 (0.01)**	0.02 (0.01)	**-0.03 (0.01)**	-0.03 (0.01)	**-0.02 (0.01)**	-0.02 (0.01)
		p	**<0.001**	<0.001	**<0.001**	<0.001	**0.016**	0.004	**<0.001**	<0.001	**0.001**	0.001
	**Ed.**	*Est (SE)*	**0.07 (0.02)**	0.08 (0.02)	0.04 (0.03)	0.04 (0.03)	**0.20 (0.03)**	0.21 (0.03)	**0.09 (0.03)**	0.10 (0.03)	**0.08 (0.03)**	0.08 (0.03)
		p	**0.001**	<0.001	0.147	0.116	**<0.001**	<0.001	**<0.001**	<0.001	**0.023**	0.016
	**Sex**	*Est (SE)*	**0.77 (0.10)**	0.78 (0.10)	**0.38 (0.12)**	0.39 (0.12)	0.22 (0.14)	0.25 (0.14)	0.07 (0.12)	0.06 (0.12)	**0.50 (0.15)**	0.51 (0.15)
		p	**<0.001**	<0.001	**.002**	0.002	0.119	0.077	0.575	0.596	**0.002**	0.001
	**APOE**	*Est (SE)*	-0.01 (0.11)	0.01 (0.11)	-0.24 (0.14)	-0.23 (0.14)	-0.08 (0.16)	-0.04 (0.16)	0.14 (0.13)	0.16 (0.13)	-0.33 (0.18)	-0.31 (0.18)
		p	.934	0.906	0.080	0.104	0.609	0.827	0.310	0.236	0.060	0.080

eTable 3. Summary of Parameter Estimates from Linear Mixed Models with Amyloid Status instead of SUVR.
Parameter estimates and standard errors are reported above for each cognitive outcome in the whole sample. The primary predictor of interest for

© 2017 Farrell ME et al. *JAMA Neurol.*

these analyses was the Amyloid Status x Time interaction, assessing whether the presence of tracer uptake above the amyloid positivity threshold (either 2SD or 3SD) was predictive of greater cognitive decline then uptake below the threshold. The Amyloid Status x Time interaction was significant for episodic memory and vocabulary, regardless of positivity threshold. Unlike the analyses with continuous SUVR, there was not a significant Amyloid Status x Time for MMSE or processing speed. Similar to the continuous SUVR analyses, we detected a significant Age x Time interaction for processing speed and reasoning, such that old age was also associated greater cognitive decline, independent of amyloid burden. Education x Time, APOE x Time, Sex x Time, and Age x SUVR x Time estimates for all cognitive variables failed to reach marginal significance and were removed from the models. Notably, the Education x Time interaction was marginally significant in the primary continuous SUVR model for MMSE, and thus the terms in the linear mixed models differ between the continuous SUVR and dichotomized amyloid status analyses. However, including or removing the Education x Time interaction did not change whether the Amyloid Status x Time interaction was significant.
[a]$p<.05$ in bold.

© 2017 Farrell ME et al. *JAMA Neurol.*

Cognitive Outcome			Episodic Memory (z)		Processing Speed (z)		Vocabulary (z)		MMSE	
	**Effect**	**Statistic**	**2SD Aβ Status Threshold**	**3SD Aβ Status Threshold**	**2SD Aβ Status Threshold**	**3SD Aβ Status Threshold**	**2SD Aβ Status Threshold**	**3SD Aβ Status Threshold**	**2SD Aβ Status Threshold**	**3SD Aβ Status Threshold**
**Effects of Time**	**Time**	*Est (SE)*	**1.46 (0.70)**	1.52 (0.91)	**1.88 (0.58)**	**2.14 (0.83)**	0.52 (0.38)	0.31 (0.60)	**2.85 (1.35)**	3.78 (2.00)
		p	**0.042**	0.109	**0.002**	**0.015**	0.183	0.612	**0.040**	0.064
	**SUVR x Time**	*Est (SE)*	**-1.33 (0.54)**	**-1.36 (0.66)**	-0.48 (0.30)	-0.49 (0.42)	-0.48 (0.30)	-0.34 (0.44)	-1.91 (1.05)	-2.52 (1.44)
		p	**0.018**	**0.048**	0.115	0.259	0.112	0.438	0.075	0.086
	**Age x Time**	*Est (SE)*	-	-	**-0.02 (0.01)**	**-0.03 (0.01)**	-	-	-	-
		p	-	-	**.001**	**0.006**	-	-	-	-
**Other Main Effects**	**SUVR**	*Est (SE)*	0.33 (0.52)	0.79 (0.63)	-0.52 (0.54)	-0.64(0.72)	**-1.72 (0.73)**	-1.37 (1.03)	-0.59 (0.78)	-0.43 (1.04)
		p	0.527	0.216	.340	0.379	**0.022**	0.277	.454	0.685
	**Age**	*Est (SE)*	**-0.03 (0.01)**	-0.02 (0.01)	**-0.04 (0.01)**	-0.03 (0.02)	0.01 (0.02)	0.01 (0.24)	-0.02 (0.01)	-0.02 (0.02)
		p	**.004**	0.109	**0.001**	0.055	0.361	0.682	0.109	0.318
	**Ed.**	*Est (SE)*	0.08 (0.05)	0.07 (0.07)	-0.03 (0.05)	-0.04 (0.09)	**0.27 (0.08)**	**0.30 (0.13)**	**0.15 (0.06)**	0.10 (0.09)
		p	0.108	0.294	.581	0.68	**0.001**	**0.025**	**.020**	0.294
	**Sex**	*Est (SE)*	**0.86 (0.21)**	**0.93 (0.29)**	0.48 (0.25)	0.62 (0.37)	0.16 (0.34)	0.10 (0.54)	**0.75 (0.28)**	0.47 (0.40)
		p	**<0.001**	**0.003**	0.61	0.103	0.652	0.862	**0.011**	0.253
	**APOE**	*Est (SE)*	-0.03 (0.21)	0.02 (0.27)	-0.11 (0.58)	-0.05 (0.34)	0.37 (0.35)	0.56 (0.51)	0.02 (0.28)	0.16 (0.38)
		p	0.902	0.944	0.671	0.895	0.288	0.277	0.952	0.684

eTable 4. Summary of Parameter Estimates from Linear Mixed Models for Amyloid Positive Adults Only.
Parameter estimates and standard errors are reported above for each cognitive outcome in amyloid positive adults, with positivity determined for both 2SD and 3SD thresholds. The primary predictor of interest, the SUVR x Time interaction, was significant for episodic memory and margrinally significant for MMSE, regardless of positivity threshold. This indicates that there is a dose-response relationship between amyloid burden and episodic memory decline within the smaller amyloid positive only sample. Unlike in the whole sample, the SUVR x Time interaction failed to reach

© 2017 Farrell ME et al. *JAMA Neurol.*

significance for processing speed or vocabulary. . Education x Time, APOE x Time, Sex x Time, and Age x SUVR x Time estimates for all cognitive variables failed to reach marginal significance and were removed from the models. [a]p<.05 in bold.

© 2017 Farrell ME et al. *JAMA Neurol.*

	Cognitive Outcome		Episodic Memory (z)		Processing Speed (z)		Vocabulary (z)		Reasoning (z)		MMSE	
	**Effect**	**Statistic**	**2SD Aβ Status**	**3SD Aβ Status**	**2SD Aβ Status**	**3SD Aβ Status**	**2SD Aβ Status**	**3SD Aβ Status**	**2SD Aβ Status**	**3SD Aβ Status**	**2SD Aβ Status**	**3SD Aβ Status**
**Effects of Time**	**Time**	*Est (SE)*	-0.60 (2.06)	0.26 (1.66)	-1.17 (1.20)	-0.40 (0.93)	1.54 (1.02)	1.04 (0.78)	0.15 (1.26)	0.82 (1.24)	2.95 (4.23)	1.69 (3.26)
		p	0.773	0.875	0.331	0.671	0.132	0.184	0.906	0.512	0.487	0.605
	**SUVR x Time**	*Est (SE)*	0.64 (2.00)	-0.21 (1.60)	1.27 (1.16)	0.61 (0.91)	-1.46 (0.99)	-0.97 (0.76)	-0.19 (1.23)	-0.32 (1.22)	-4.31 (4.15)	-2.80 (3.14)
		p	0.751	0.898	0.274	0.508	0.142	0.201	0.876	0.791	0.302	0.375
	**Age x Time**	*Est (SE)*	-	-	-0.01 (0.00)	**-0.01 (0.00)**	-	-	-	-0.01 (0.00)	-	-
		p	-	-	0.044	**0.01**	-	-	-	0.059	-	-
	**Ed. x Time**	*Est (SE)*	-	-	-	-	-	-	-	-	**0.14 (0.06)**	**0.12 (0.06)**
		p	-	-	-	-	-	-	-	-	**0.039**	**0.040**
**Other Main Effects**	**SUVR**	*Est (SE)*	-0.82 (1.90)	-0.82 (1.52)	0.09 (2.07)	0.10 (1.64)	**4.32 (2.18)**	**3.89 (1.80)**	**5.23 (1.99)**	**3.41 (1.59)**	6.37 (3.36)	4.24 (2.60)
		p	0.668	0.589	0.967	0.95	**0.049**	**0.024**	**0.009**	**0.034**	0.059	0.105
	**Age**	*Est (SE)*	**-0.01 (0.00)**	**-0.02 (0.01)**	**-0.04 (0.01)**	**-0.04 (0.01)**	**0.02 (0.01)**	**0.02 (0.01)**	**-0.04 (0.01)**	**-0.04 (0.01)**	**-0.02 (0.01)**	**-0.02 (0.01)**
		p	**0.006**	**0.001**[a]	**<0.001**	**<0.001**	**.011**	**0.005**	**<0.001**	**<0.001**	**0.004**	**0.002**
	**Ed.**	*Est (SE)*	**0.07 (0.03)**	**0.08 (0.02)**	**0.08 (0.03)**	**0.06 (0.03)**	**0.18 (0.03)**	**0.19 (0.03)**	**0.10 (0.03)**	**0.10 (0.03)**	-0.00 (0.05)	0.02 (0.05)
		p	**0.005**	**0.001**	**0.012**	**0.029**	**<0.001**	**<0.001**	**0.001**	**0.001**	0.932	0.687

© 2017 Farrell ME et al. *JAMA Neurol.*

	**Sex**	*Est (SE)*	**0.74 (0.11)**	**0.75 (0.11)**	**0.29 (0.14)**	**0.30 (0.13)**	0.26 (0.15)	0.26 (0.14)	0.03 (0.13)	-0.02 (0.12)	**0.43 (0.18)**	**0.49 (0.17)**
		p	**<0.001**	**<0.001**	**0.039**	**0.022**	0.080	0.066	0.800	0.866	**0.021**	**0.006**
	**APOE**	*Est (SE)*	0.02 (0.14)	0.003 (0.13)	**-0.39 (0.17)**	**-0.34 (0.16)**	-0.18 (0.18)	-0.19 (0.17)	0.11 (0.16)	0.12 (0.15)	-0.41 (0.23)	**-0.45 (0.21)**
		p	0.903	0.979	**0.024**	**0.033**	0.330	0.251	0.483	0.415	0.073	**0.034**

eTable 5. Summary of Parameter Estimates from Linear Mixed Models for Amyloid Negative Adults Only.
Parameter estimates and standard errors are reported above for each cognitive outcome in amyloid negative adults, with negativity determined for both 2SD and 3SD thresholds. . There were no significant SUVR x Time interactions for any cognitive variable regardless of threshold, suggesting relatively high SUVR within the amyloid negative range is not associated with cognitive decline. However, there was a significant Age x Time interaction for processing speed (and a marginal significant Age x Time for reasoning), such that old age was predictive of greater processing speed decline, independent of amyloid burden (see eFigure 3). There was also a significant Education x Time interaction for MMSE, such that increasing education was associated with more positive change in MMSE. APOE x Time, Sex x Time, and Age x SUVR x Time estimates for all cognitive variables failed to reach marginal significance and were removed from the models. [a]p<.05 in bold.

© 2017 Farrell ME et al. *JAMA Neurol.*

Cognitive Outcome		Episodic Memory (z)		Processing Speed (z)		Vocabulary (z)		Reasoning (z)		MMSE	
		Est (SE)	***p***	***Est (SE)***	***p***	***Est (SE)***	***p***	***Est (SE)***	***p***	***Est (SE)***	***p***
**Effects of Time**	**Time**	2.87 (1.73)	0.103	-1.34 (1.00)	0.188	**2.18 (0.89)**	**0.019**	-0.40 (1.04)	0.704	-3.83 (3.35)	0.26
	**SUVR x Time**	-2.63 (1.65)	0.118	1.08 (0.96)	0.263	**-2.05 (0.86)**	**0.021**	0.38 (1.00)	0.705	4.32 (3.20)	0.184
**Other Main Effects**	**SUVR**	-0.54 (1.72)	0.754	1.28 (2.05)	0.537	1.74 (2.24)	0.441	0.06 (1.93)	0.974	-0.04 (2.96)	0.988
	**Age**	0.02 (0.02)	0.315	-0.03 (0.02)	0.263	0.03 (0.03)	0.239	-0.04 (0.02)	0.117	-0.04 (0.03)	0.224
	**Ed.**	**0.10 (0.04)**	**0.038**[a]	0.06 (0.06)	0.281	**0.17 (0.06)**	**0.009**	0.07 (0.05)	0.181	0.10 (0.07)	0.148
	**Sex**	**0.83 (0.19)**	**<0.001**	0.03 (0.25)	0.903	0.43 (0.27)	0.122	0.23 (0.23)	0.324	0.29 (0.31)	0.352
	**APOE**	-0.10 (0.19)	0.579	0.14 (0.24)	0.575	-0.22 (0.27)	0.413	0.01 (0.29)	0.95	-0.61 (0.30)	0.051

eTable 6. Summary of Parameter Estimates from Linear Mixed Models for Middle-Aged Adults Only.
Parameter estimates and standard errors are reported above for each cognitive outcome in the middle-aged subsample (n=51). There was a significant SUVR x Time interaction for vocabulary, such that increasing SUVR predicted increasingly negative change. The SUVR x Time interaction failed to reach statistical significance for any other cognitive variable. There was also a significant main effect of time for vocabulary, with individuals showing improvement over time. Age x Time, Education x Time, APOE x Time, Sex x Time, and Age x SUVR x Time estimates for all cognitive variables failed to reach marginal significance and were removed from the models. [a]p<.05 in bold.

© 2017 Farrell ME et al. *JAMA Neurol.*

Cognitive Outcome		Episodic Memory (z)		Processing Speed (z)		Vocabulary (z)		Reasoning (z)		MMSE	
		Est (SE)	***p***	***Est (SE)***	***p***	***Est (SE)***	***p***	***Est (SE)***	***p***	***Est (SE)***	***p***
**Effects of Time**	**Time**	***1.06 (0.43)***	***0.016***[a]	***1.26 (0.42)***	***0.003***	***0.50 (0.22)***	***0.024***	***1.72 (0.63)***	***0.007***	***2.33 (0.89)***	***0.01***
	**SUVR x Time**	***-1.03 (0.38)***	***0.008***	***-0.53 (0.22)***	***0.019***	***-0.46 (0.19)***	***0.019***	-0.29 (0.33)	0.389	***-1.63 (0.79)***	***0.041***
	**Age x Time**	-	-	***-0.02 (0.01)***	***0.005***	-	-	***-0.02 (0.01)***	***0.009***	-	-
**Other Main Effects**	**SUVR**	-0.25 (0.38)	0.509	-0.56 (0.40)	0.173	***-0.92 (0.47)***	***0.049***	-0.82 (0.42)	0.053	-0.19 (0.64)	0.77
	**Age**	***-0.02 (0.01)***	***0.017***	***-0.04 (0.01)***	***<0.001***	0.001 (0.01)	0.912	***-0.02 (0.01)***	***0.017***	***-0.03 (0.01)***	***0.012***
	**Ed.**	***0.07 (0.03)***	***0.005***	0.05 (0.03)	0.137	***0.22 (0.04)***	***<0.001***	***0.10 (0.03)***	***0.001***	***0.08 (0.03)***	***0.04***
	**Sex**	***0.73 (0.12)***	***<0.001***	***0.46 (0.14)***	***0.002***	0.17 (0.17)	0.312	0.01 (0.14)	0.969	***0.58 (0.19)***	***0.002***
	**APOE**	-0.05 (0.15)	0.729	0.29 (0.17)	0.104	-0.11 (0.20)	0.58	-0.23 (0.17)	0.193	0.09 (0.23)	0.682

eTable 7. Summary of Parameter Estimates from Linear Mixed Models for Older Adults Only.

Parameter estimates and standard errors are reported above for each cognitive outcome in the older adult subsample (n=123). Results were very similar to those for the larger sample aged 40-89, suggesting the whole sample effects were primarily driven by the older adults. The SUVR x Time interaction was significant for episodic memory, processing speed, vocabulary and MMSE, indicating a significant dose-response relationship between baseline amyloid burden and cognitive change (see eFigure5). There was also a significant positive main effect of Time for episodic memory, processing speed, vocabulary, reasoning and MMSE, indicating that there was an increase over time in these variables independent of amyloid burden. Next, we detected a significant Age x Time interaction for processing speed and reasoning, such that old age was associated with greater cognitive decline, independent of amyloid burden. The Age x Time interaction failed to reach marginal significance for the remaining cognitive variables are was removed from the models. Education x Time, APOE x Time, Sex x Time, and Age x SUVR x Time estimates for all cognitive variables failed to reach marginal significance and were removed from the models. [a]p<.05 in italics.

© 2017 Farrell ME et al. *JAMA Neurol.*
